# Difficult diagnosis of brainstem glioblastoma multiforme in a woman: a case report and review of the literature

**DOI:** 10.1186/1752-1947-3-87

**Published:** 2009-10-30

**Authors:** Shaheen E Lakhan, Lindsey Harle

**Affiliations:** 1 Global Neuroscience Initiative Foundation, Los Angeles, CA, USA

## Abstract

**Introduction:**

Brainstem gliomas are rare in adults. They most commonly occur in the pons and are most likely to be high-grade lesions. The diagnosis of a high-grade brainstem glioma is usually reached due to the presentation of rapidly progressing brainstem, cranial nerve and cerebellar symptoms. These symptoms do, however, overlap with a variety of other central nervous system disorders. Magnetic resonance imaging is the radiographic modality of choice, but can still be misleading.

**Case presentation:**

A 48-year-old Caucasian woman presented with headache and vomiting followed by cerebellar signs and confusion. Magnetic resonance imaging findings were suggestive of a demyelinating process, but the patient failed to respond to therapy. Her condition rapidly progressed and she died. At autopsy, a high-grade invasive pontine tumor was identified. Histological evaluation revealed glioblastoma multiforme.

**Conclusion:**

While pontine gliomas are rare in adults, those that do occur tend to be high-grade and rapidly progressive. Progression of symptoms from non-specific findings of headache and vomiting to rapid neurological deterioration, as occurred in our patient, is common in glioblastoma multiforme. While radiographic findings are often suggestive of the underlying pathology, this case represents the possibility of glioblastoma multiforme presenting as a deceptively benign appearing lesion.

## Introduction

Brainstem gliomas are rare in adults, with approximately 100 cases reported per year [1]. The majority of tumors occur in the pons, and in this location tumors are most commonly high-grade. The clinical presentation is variable, depending upon the exact location and growth rate of the lesion. Diagnosis can be difficult, with the differential including infectious, inflammatory, autoimmune or vasculitic disease. Here we report the case of a patient with glioblastoma multiforme (GBM) that was initially misdiagnosed as sinusitis, tension headache, myasthenia gravis and demyelinating disease. The correct diagnosis was not reached until autopsy.

## Case presentation

A 48-year-old otherwise apparently healthy Caucasian woman presented to our clinic complaining of headache, nausea and vomiting for 5 days. Physical examination and laboratory tests were within normal limits. She was diagnosed with tension headache and sinusitis and discharged on trimethoprim-sulfamethoxazole. The patient returned to the clinic 3 days later with resolution of her headache but continued vomiting, approximately twice per day. She was treated with an over-the-counter proton pump inhibitor for suspected gastroesophageal reflux.

Over the following week, she developed ataxia, diplopia and recurrence of her headache. She presented to the emergency department, at which time a T2-weighted magnetic resonance image (MRI) scan of the head showed increased signaling of the left temporal lobe, bilateral pontine areas, left peridentate nucleus and cervical spinal cord. These findings were thought to represent an acute demyelinating process. At this point, clinical suspicion for myasthenia gravis was high, and she was administered a trial of pyridostigmine without improvement.

During her hospitalization, she developed a blood pressure of 190/90 mmHg, a serum sodium level of 123 mEq/l, a glucose level of 176 mg/dl, and a white blood cell count of 12,800 cells/ml. Clinical examination during her stay showed progression of neurological symptoms with decreased sensorium, hallucinations, decreased attention span, and gait disturbance. A lumbar puncture showed increased opening pressure. Based on the radiographic, laboratory and clinical findings, a diagnosis of acute demyelinating encephalopathy was made; suspicions of infectious encephalitis, collagen vascular disease, and toxic encephalitis were high. She received plasmapheresis without improvement.

A follow-up T2-weighted MRI was performed, which showed increasing enlargement of the bilateral pontine regions with hydrocephalus and mass effect on the fourth ventricle. Because of the absence of pathological enhancement of the pons, this was felt to be an aggressive demyelinating process or a low-grade glioma. The patient continued to deteriorate neurologically, became comatose and died approximately 4 weeks after admission.

**Figure 1:**
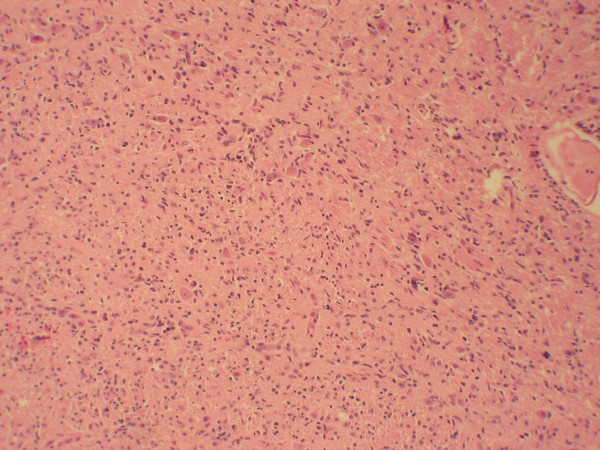
**Pons lesion, low power**. The tumor completely effaced the normal pontine architecture. Tumor cells invaded into the cerebellum and cervical spinal cord (not shown).

**Figure 2:**
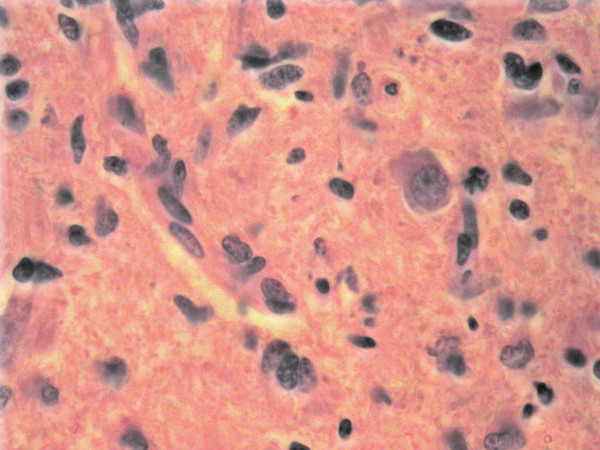
**Pons lesion, high power**. The tumor is composed of pleomorphic, poorly differentiated glial cells. Immunohistochemical staining for Ki-67 was diffusely positive (not shown), consistent with the rapid progression seen clinically.

At autopsy, the brain was edematous with a weight of 1375 g. The pons and medulla showed an infiltrative mass with extension into the cerebellum. Histological examination revealed a poorly differentiated infiltrative astrocytoma with nuclear pleomorphism, increased mitotic activity and focal necrosis (Figures 1 and 2). Staining for glial fibrillary acidic protein (GFAP) and MIB-1 were diffusely positive. The tumor microscopically extended into the middle cerebellar peduncles and the upper cervical spinal cord.

## Discussion

The clinical presentation of brainstem gliomas is often non-specific and misleading. Radiological imaging is imperative to demonstrate a brainstem lesion but, as seen in this patient, is not always capable of detecting the true nature of the lesion. Based on the signaling pattern, our patient's MRI abnormalities were thought to represent a diffuse demyelinating pattern. On subsequent MRI, an enlarging brainstem mass was identified but was felt to represent an area of demyelination or, at most, a low-grade glioma. Autopsy confirmed the diagnosis of GBM with high-grade and infiltrative features.

Although they account for 30% of pediatric posterior fossa tumors [2], gliomas of the brainstem are extremely rare in adults, with approximately 100 adult cases reported each year [1]. The majority of brainstem gliomas occur in the pons, where they are more likely to be high grade and have a poor prognosis [3]. These tumors progress rapidly, invading adjacent structures and resulting in an average survival of less than 1 year [4]. Due to the accelerated growth rate, symptoms of high-grade pontine gliomas are usually short in duration and rapidly progressive in severity.

While histological examination is required for definitive diagnosis, due to the difficult nature of obtaining tissue from the brainstem, radiographic imaging is often the preferred method of diagnosis and classification [5]. Computed tomography (CT) of high-grade pontine gliomas typically shows a hypodense or isodense lesion; MRI shows a hypointense lesion on T1-weighted images and a hyperintense lesion on T2-weighted images. Rapid diffusion MRI, thallium single photon emission computed tomography (SPECT) [6] and positron emission tomography (PET) [7] are emerging as potentially superior imaging techniques for brainstem lesions. The differential diagnosis of a suspected high-grade glioma visualized radiographically is broad and includes vascular malformation, encephalitis, parasitic infection, demyelinating disorder, and hamartomas.

Misdiagnoses associated with brainstem gliomas are reported in the literature. Cases have been mistaken for cervical myelopathy [8], meningitis [9] and toxoplasmosis [10]. This is in part due to the common symptomatology seen in various lesions of the central nervous system. Most physicians rarely encounter this entity, if at all, and there are few analyses in the published literature.

Badhe *et al. *performed a retrospective analysis of 45 cases of brainstem gliomas [11]; 24% occurred in patients over the age of 20. Fifteen percent were grade IV; most were located in the pons (55.55%), followed by the medulla (31.01%) and the midbrain (13.33%). The presenting features were variable but predictable given the location of the lesion, including cranial nerve palsies, cerebellar signs, headache, papilledema, hydrocephalus, distal weakness and sensory loss. Mantravadi *et al*. reported an autopsy series of 25 brainstem gliomas [12]. GBM was the most common neoplasm (48%) and the pons was the most common location. Pontine tumors were more likely to invade superiorly and laterally into adjacent structures than tumors of the medulla or midbrain.

## Conclusion

The ability to recognize brainstem tumors early in the disease process is imperative to prevent mass effect on surrounding structures, which can be rapidly fatal. Radiographic imaging by MRI is the most accurate tool for initial evaluation but can be misleading, as low-grade tumors, inflammatory diseases and demyelinating processes can have a similar appearance to high-grade lesions. Likewise, the clinical signs of brainstem tumors overlap with a variety of other central nervous system diseases. The rarity of brainstem gliomas in adults makes recognition all the more difficult. In order to provide rapid and early treatment, a high index of suspicion must be maintained for new onset of brainstem and cerebellar symptoms that fail to respond to treatment for more common disorders.

## Consent

Written informed consent was obtained from the next-of-kin of the patient for publication of this case report and any accompanying images. A copy of the written consent is available for review by the Editor-in-Chief of this journal.

## Competing interests

The authors declare that they have no competing interests.

## Authors' contributions

SL and LH secured the case, conducted the literature review, and participated in the preparation of the manuscript. Both authors read and approved the final manuscript.
